# Supplementary material for: The transition to agricultural cultivation of neo-crops may fail to account for wild genetic diversity patterns: insights from the Cape Floristic Region
Source: PeerJ. 2021 Jun 9;9:e11462. doi: 10.7717/peerj.11462 (PMC8197031; doi:10.7717/peerj.11462)
Supplement: Supplemental Information 1 [file peerj-09-11462-s001.doc]

The transition to agricultural cultivation of neo-crops may fail to account for wild genetic diversity patterns: insights from the Cape Floristic Region

## Appendix S1: PCR and HRM protocol

Table S1: Polymerase Chain Reaction primers used to amplify the parent region of the loci screened by HRM. Primers in bold were used for unidirectional sequencing.

Table S2: PCR amplification protocol for the primers sources from Shaw et al. 2007. See Table A1 for annealing temperatures. See

Table S3: Cyclopia specific primers used for haplotype detection via HRM analysis.
